# Global sensitivity analysis of a single-cell HBV model for viral dynamics in the liver

**DOI:** 10.1016/j.idm.2021.10.003

**Published:** 2021-10-27

**Authors:** Md Afsar Ali, S.A. Means, Harvey Ho, Jane Heffernan

**Affiliations:** a York University, Canada; b Massey University, New Zealand; c The University of Auckland, New Zealand

**Keywords:** Latin hypercube sampling, Partial rank correlation coefficient (PRCC), Sobol method, HBV, Liver

## Abstract

The predictive accuracy of mathematical models representing anything ranging from the meteorological to the biological system profoundly depends on the quality of model parameters derived from experimental data. Hence, robust sensitivity analysis (SA) of these critical model parameters aids in sifting the influential from the negligible out of typically vast parameter regimes, thus illuminating key components of the system under study. We here move beyond traditional local sensitivity analysis to the adoption of global SA techniques. Partial rank correlation coefficient (PRCC) based on Latin hypercube sampling is compared with the variance-based Sobol method. We selected for this SA investigation an infection model for the hepatitis-B virus (HBV) that describes infection dynamics and clearance of HBV in the liver [Murray & Goyal, 2015]. The model tracks viral particles such as the tenacious and nearly ineradicable covalently closed circular DNA (cccDNA) embedded in infected nuclei and an HBV protein known as p36. Our application of these SA methods to the HBV model illuminates, especially over time, the quantitative relationships between cccDNA synthesis rate and p36 synthesis and export. Our results reinforce previous observations that the viral protein, p36, is by far the most influential factor for cccDNA replication. Moreover, both methods are capable of finding crucial parameters of the model. Though the Sobol method is independent of model structure (e.g., linearity and monotonicity) and well suited for SA, our results ensure that LHS-PRCC suffices for SA of a non-linear model if it is monotonic.

## 1 Introduction

Liver infection with hepatitis B is a life-threatening disease–causing hepatocellular carcinoma, liver cirrhosis, liver damage and failure with an estimated death total of at least 887, 000 worldwide each year (Elgouhari et al., 2008; Tarao et al., 2019; World Health Organization, 2015). About 250 million people worldwide suffer from chronic hepatitis B virus (HBV) infection, and few effective treatment options exist (Aparna et al., 2015; Means et al., 2020).

A better understanding of HBV infection in-host is needed to better inform pharmaceutical intervention. The discovery of effective treatments critically hinges on the characterization of the HBV infection and replication dynamics within hepatocytes, cells in the liver (Fausto & Campbell, 2003). HBV virus replication requires the development of closed covalent circular DNA (cccDNA) from the viral DNA that is injected into the cell upon infection (Guo & Guo, 2015). During infection, cccDNA accumulates in the cell nuclei in which it persists as a stable episome and functions as a template for the transcription of viral genes. Chronic HBV infection is maintained in cells by a replicative form of HBV cccDNA (Liu & Xin, 2019; Shi & Shi, 2009). As such, HBV cccDNA has been identified as a potential and important target for therapeutic intervention, and represents a focus for antiviral drug discovery.

In a recent study of HBV infection, Murray and Goyal (Murray & Goyal, 2015) developed a model of HBV replication in a single hepatocyte. The model explicitly considers cccDNA, and it tracks intracellular and extracellular virus particles. The model has since been extended to study cell-cell transmission (Goyal & Murray, 2016) and has been extended to a study of a hepatocyte-sinusoid model (Cangelosi et al., 2017). As such, the single-cell model developed by Murray and Goyal (Murray & Goyal, 2015) has been shown to provide an important building block over which an HBV infection model of a liver could be constructed. The model thus also provides an important base to study potential drug targets.

Herewith, we conduct a sensitivity analysis on the Murray and Goyal model (Murray & Goyal, 2015). We apply two global SA techniques, LHS PRCC (Blower & Dowlatabadi, 1994; Marino et al., 2008; Zheng & Rundell, 2006), and a variance-based Sobol method (Sobol’, 1990; Sobol’, 2001) to identify key parameters that most drive HBV replication in a liver cell. We pay particular attention to infection processes involving cccDNA. The outcomes of our study are two-fold. Our SA results can be used to determine key parameters that affect cccDNA, which can, in turn, be used to inform drug therapy development. Additionally, the SA results can be used to inform the future construction of a spatial model of the liver from the Murray and Goyal model building block.

## 2 Overview of sensitivity analysis, computing methods and mathematical model

### 2.1 Uncertainty, sensitivity and scale

**Fig. 1:**
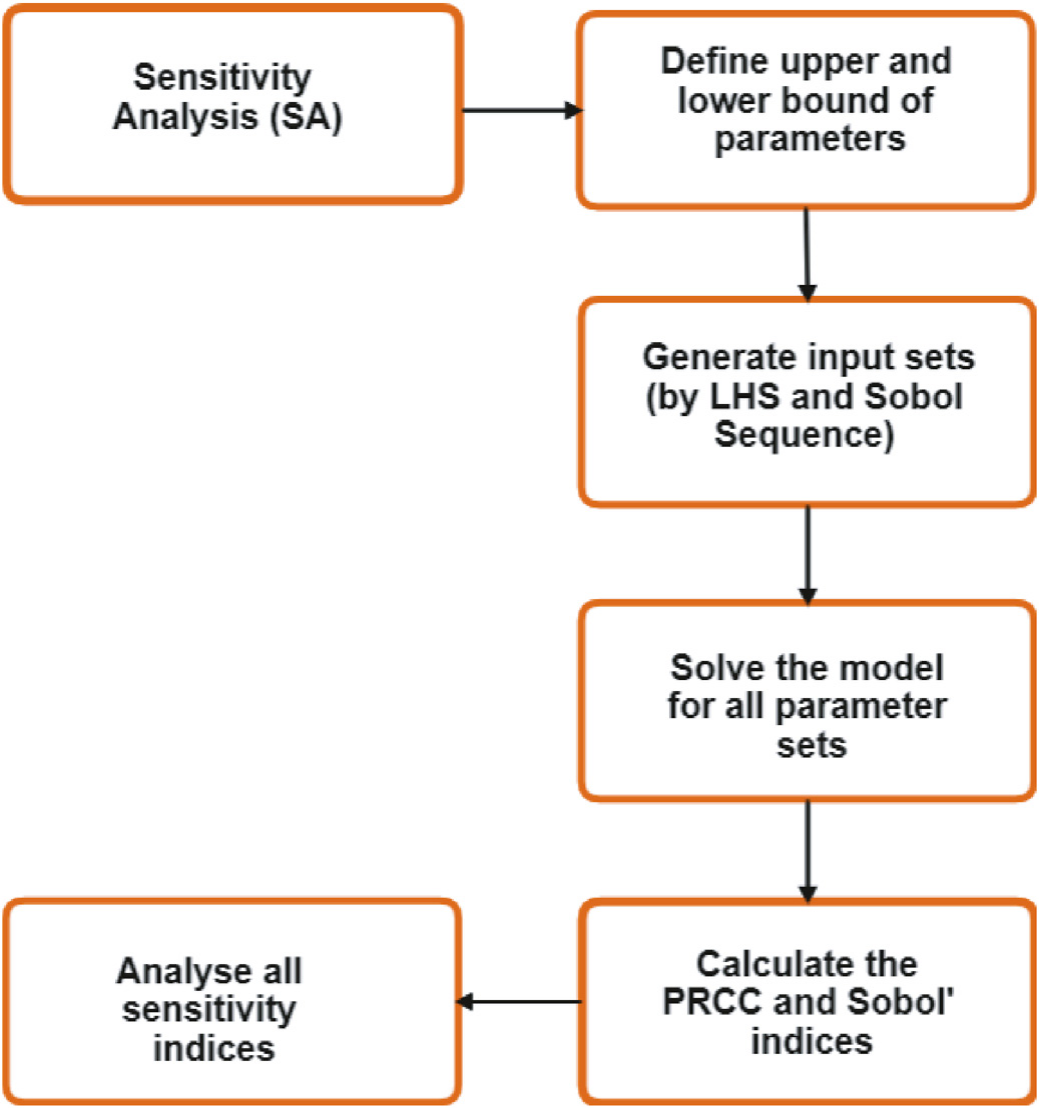
Working steps of sensitivity analysis are listed concisely in the diagram, and described below: (a) Choose a sensitivity analysis method based on the number of model evaluations, the correlation structure of input-output parameters and an experimental design. (b) Identify simulation input-output parameters of interest and what input factors are needed to include in the analysis. (c) Define upper and lower bound of input parameters by taking respectively 15 − 20*%* more and less from the mean value of each parameter and generate input sets of parameters by LHS method and Sobol sequence. An input set has the form of *N* strings of input factor values on which the model is evaluated. (d) Evaluate the model on the generated input samples and produce the outputs, which contain *N* output values in the desired form. (e) Analyse the model outputs and draw conclusions depending on PRCC and Sobol indices calculated using the outputs. (f) Based on the observation of model outputs parameter sample size is determined. More detail on sample size can be found in Appendix D.

Uncertainty in model parameters exists. While estimates of some model parameters may exist, there is no true measurement of a parameter that is universal. Uncertainty in model parameters will affect confidence in predictions of mathematical models. Uncertainty analysis (UA) aims at providing a measurable degree of confidence to address this complication (Marino et al., 2008). Falling under this umbrella of uncertainty, is the sensitivity of model outcomes to model parameters (Helton & Davis, 2002; Helton & Oberkampf, 2004). Sensitivity analysis (SA) itself can assist in efficient parameter calibration and fortify models against over-parameterization (Van Griensven et al., 2005) – further relieving modelers of excessive effort. Two scales of SA emerge on both a local (LSA) and global (GSA) (Van Griensven et al., 2005; Zhan et al., 2013) range. Whereas LSA aims at individual parameter influences, GSA grapples with entire regions for families of parameters and quantification of their interactions on output variables (Zi, 2011). Given likely multitudes of dimensions to parameter spaces and constraints of input-output realism for model predictions, SA methods are an essential addition to a modeler's toolkit for refinement and simplicity. We here consider two GSA approaches mentioned: LHS-PRCC and the variance-based Sobol method, which are briefly described in turn here, and provide modestly more detail in the Appendix [A, B, C]. Substantial introductions to these methods are available where we refer the reader as noted. General working steps of sensitivity analysis are listed concisely in the diagram presented in Fig. 1.

### 2.2 Latin hypercube sampling-LHS

Introduced by Mckay et al. (Helton & Davis, 2003; Iman & Conover, 1982; Iman et al., 1981; McKay et al., 1979), LHS generates samples of model inputs arrayed across a ‘hypercube’ whose dimension corresponds to the number of model parameters; call this dimension *p*. Utilizing parameter ranges partitioned into intervals, LHS deploys a selected probability density function for sampling parameter values from within these intervals that are then paired (in *p*-dimensional tuples) with samples, in like manner, for the entire suite of parameters. Simulations of the model are then performed iterating over all the *p*-sized parameter tuples. Depending on the number of interval partitions, this can result in a substantial number of *p*-sized parameter suites for testing – but vastly fewer than required for interrogating the entire parameter space.

Results of simulations across the LHS-sampled space are compared for influence with Pearson correlations – here a ‘Partial rank correlation coefficient’ (PRCC). Resulting correlations may be negative to positive in the range [-1, 1] as is usual (Marino et al., 2008) – illuminating the influence of parameters as either amplifying or dampening model outputs. Generally, PRCC analysis provides a measurement of the nonlinear, but monotonic, relationship between a model output and the model parameters. Examples in disease modelling can be found in (Blower & Dowlatabadi, 1994; Marino et al., 2008; Zheng & Rundell, 2006), and many other studies.

### 2.3 Sobol method

Alternatively, the output variables of a nonlinear model are amenable to Analysis of Variances used with the Sobol technique (Sobol’, 1990; Sobol’, 2001). An orthogonal decomposition of the model into components for each input parameter is assembled across a *p* − dimensional normalized hypercube, *I*^*p*^, with *I* over [0,1] and *p* again the dimension of parameter space. With such a decomposition of the model combined with a uniform sampling of the *p*-dimensional hypercube, variances per model output are computed. These variances are in turn utilized for calculation of the Sobol index – a metric of influence for a given parameter. Two such indices are computed revealing the individual and the total influence of a parameter, denoted *S*_*i*_ and *ST*_*i*_, whose values fall between [0, 1]. When, for instance, *S*_*i*_ = *ST*_*i*_ = 0, the parameter influence over the model is nil, but, by contrast, supreme when *S*_*i*_ = *ST*_*i*_ = 1. Typically, Sobol indices with values greater than 0.05 are considered significant. Unlike the PRCC results, however, Sobol coefficients do not indicate positive or negative influence but merely significance.

### 2.4 Single-cell model of hepatitis B viral dynamics in the liver

**Fig. 2:**
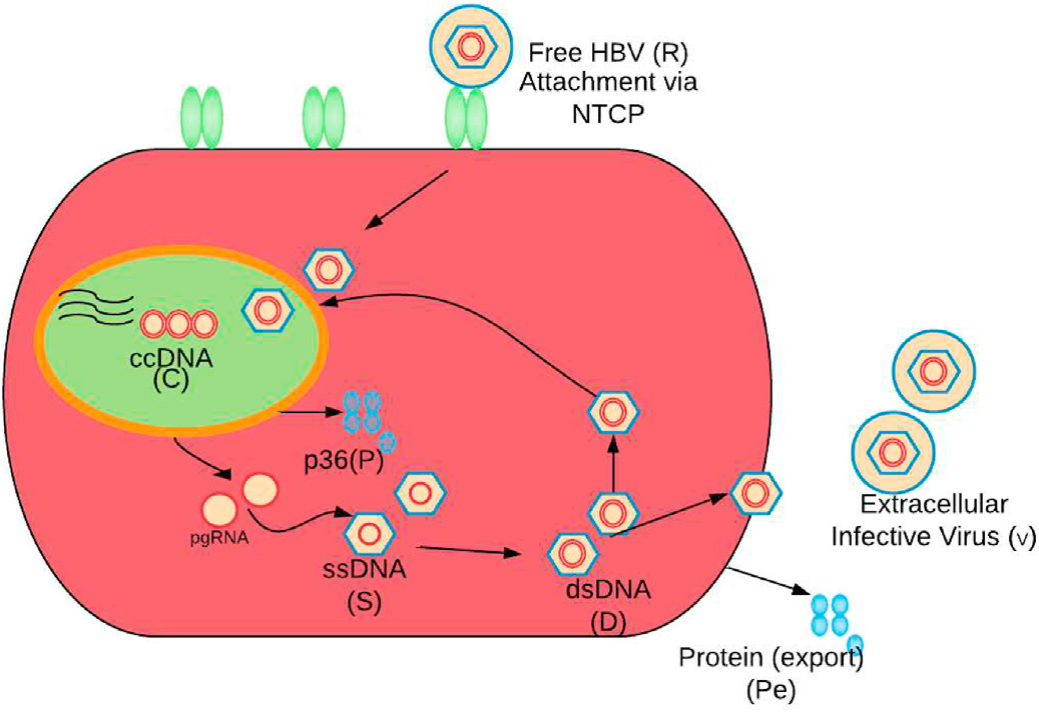
The schematic shows the HBV replication cycle in a single cell. HBV enters the hepatocyte from outside of the cell through NTCP, denoted here as variable R, and the genome (cccDNA) is transferred to the nucleus, labeled as C. By the transcription of cccDNA, protein (p36) and pre-genomic RNA (pgRNA) are produced. These are cytoplasmic pgRNA and are packaged with polymerase and envelope proteins into nucleocapsids, and by the reverse transcription, RNA is converted into DNA. In this process, first single-stranded DNA (ssDNA), labeled S, and then double-stranded DNA (dsDNA), denoted D, are produced. Depending upon the level of p36 proteins in the cytoplasm, labeled P, dsDNA will either return to the nucleus or will be released as complete infective HBV virions labeled V outside of the cell to infect other hepatocytes in the liver.

We performed global sensitivity analysis of a HBV model (Murray & Goyal, 2015) representative of single-cell viral infection dynamics in the liver (see Fig. 2). HBV invades hepatocytes via the NTCP and establishes the stable covalently-closed circular viral DNA (cccDNA) in the nucleus. Subsequent transcriptions are two intermediate forms such as the single-stranded DNA (ssDNA) and the dual-strand DNA (dsDNA). Of the milieu of viral proteins produced by HBV, the model tracks *p36* whose action determines whether the dsDNA intermediate continues to a complete HBV and is released, or instead reinforces the nucleic cccDNA pool. In the model, the intracellular numbers of ssDNA, dsDNA, and infecting rcDNA are respectively denoted by *S*, *D*, and *R*. The number of cccDNA copies in the cell is denoted by *C*; the number of protein (p36) molecules inside and outside of the cell are respectively denoted by *P* and *P*_*E*_, and the number of virions in serum originated from the cell is denoted by *V*. The intra-cellular viral replication dynamics in a single cell, shown in Fig. 2, is modelled by the following set of time-delayed differential equations:

(1) $$\left\{\begin{array}{l} \frac{dR}{dt}=kV\left(t-\tau \right)-\mu_{R}R-b_{R}e^{\left(-\lambda P\right)}R\;\\ \frac{dC}{dt}=b_{R}e^{-\lambda P\left(t-\tau \right)}R\left(t-\tau \right)+b_{D}e^{-\lambda P\left(t-\tau \right)}D\left(t-\tau \right)-\mu C\;\\ \frac{dS}{dt}=aC\left(t-\tau \right)-b_{S}S\;\\ \frac{dD}{dt}=b_{S}S\left(t-\tau \right)-b_{D}D\;\\ \frac{dP}{dt}=a_{P}C\left(t-\tau \right)-b_{P}P\;\\ \frac{dP_{E}}{dt}=b_{P}P\left(t-\tau \right)-c_{P_{E}}P_{E}\;\\ \frac{dV}{dt}=b_{D}\left(1-e^{-\lambda P\left(t-\tau \right)}\right)D\left(t-\tau \right)-c_{V}V\; \end{array}\right)$$

Here, *k* is a rate at which virions infect a cell and produce rcDNA which is lost at a rate *μ*_*R*_; depending on the level of p36, rcDNA is transported to the nucleus at a rate *b*_*R*_ and there at the same rate rcDNA is converted to cccDNA which is lost at a rate *μ*; cccDNA is converted to ssDNA at a rate *a* and is lost at a rate *b*_*S*_; ssDNA is converted to dsDNA at a rate *b*_*S*_ and is also lost at a rate *b*_*D*_; *a*_*P*_ is the rate of synthesis of protein p36 which is lost at a rate *b*_*p*_; protein (p36) is exported outside of the cell at a rate *b*_*P*_ and lost at a rate *c*_*PE*_, and virions are released from the cell at a rate *b*_*D*_ and lost in serum at a rate *c*_*V*_. *λ* maintains the average level of p36 that directs *R* and *D* to nucleus, and *V* to the outside of the cell. The time delay, *τ*, is considered as 30 min in a day for all simulations. The parameter names with some symbolic modifications without changing their meaning and values are adopted from the original paper published by Murray and Goyal (Murray & Goyal, 2015). The list of parameters with their mean values and ranges used for the generation of parameter samples for SA is given in Table 1.

**Table 1:** Model parameters used in the simulation are taken predominantly from those used by Murray and Goyal (Murray & Goyal, 2015) with a slight symbolic modification for the visualization of outputs corresponding to the respective parameters with a clear distinguish-ability. As we do not have realistic ranges for all parameters, instead of varying some in realistic ranges, and others over wide ranges, we choose to vary all in wide ranges from 50 to 150%. Using a wide range for each parameter, we have a big parameter space to compare Sobol and LHS-PRCC methods.

Parameter	Symbol (in (Murray & Goyal, 2015))	Symbol (in our study)	Value ($\frac{1}{\text{day}}$)	Reference
Cell infection rate	*k*	*k*	0.3	(Cangelosi et al., 2017; Murray & Goyal, 2015)
cccDNA synthesis rate	*b*	*b*_*R*_	*log*(2)	(Cangelosi et al., 2017; Murray & Goyal, 2015)
Conversion rate of cccDNA to ssDNA	*a*	*a*	50	(Cangelosi et al., 2017; Murray & Goyal, 2015)
Conversion rate of ssDNA to dsDNA	*b*	*b*_*S*_	*log*(2)	(Cangelosi et al., 2017; Murray & Goyal, 2015)
*P*36 synthesis rate	*a*_*P*_	*a*_*P*_	1000 × *a*	(Cangelosi et al., 2017; Murray & Goyal, 2015)
[1ex] *P*36 exporting rate	*b*_*P*_	*b*_*P*_	*log*(2)	(Cangelosi et al., 2017; Murray & Goyal, 2015)
dsDNA transportation rate to the nucleus/Virions release rate	*b*	*b*_*D*_	*log*(2)	(Cangelosi et al., 2017; Murray & Goyal, 2015)
rcDNA degradation rate	*μ*_*R*_	*μ*_*R*_	*log*(2)	(Cangelosi et al., 2017; Murray & Goyal, 2015)
cccDNA degradation rate	*μ*	*μ*	*log*(2)/50	(Cangelosi et al., 2017; Murray & Goyal, 2015)
				
p36 influence on *R* and *dsDNA* → *C*, and on *R* to export *V*	*λ*	*λ*	1/100000	(Cangelosi et al., 2017; Murray & Goyal, 2015)
				
p36 degradation rate	*c*	$c_{P_{E}}$	24 × *log*(2)/4	(Cangelosi et al., 2017; Murray & Goyal, 2015)
Virions degradation rate	*c*	*c*_*V*_	24 × *log*(2)/4	(Cangelosi et al., 2017; Murray & Goyal, 2015)
Time delay	*τ*	*τ*	30/1440	(Cangelosi et al., 2017; Murray & Goyal, 2015)

The model in Eq. (1) is completed by adding initial conditions. For all simulations, we use initial conditions for all populations as: (*R*, *C*, *S*, *D*, *P*, *P*_*E*_, *V*) = (0, 0, 0, 0, 0, 0, 1). Solving the model and performing the sensitivity analysis is completed as described in Appendix E “Computational Aspects”.

## 3 Results

### 3.1 Time-evolution of cccDNA population

**Fig. 3:**
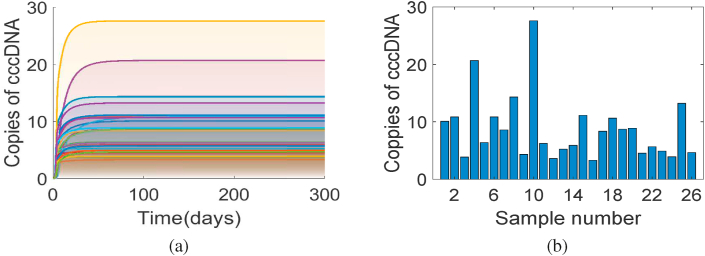
Simulation runs are carried out for all samples of size *N* = 5200 and the time evolution of all cccDNA curves is shown in Appendix F. For visibility, a few cccDNA curves are plotted showing the evolution of cccDNA copies over time in Figure (a). In Figure (b), for each of these cccDNA curves, the number of cccDNA at *t* = 300(*days*) is plotted to show the variation of cccDNA copies for different sets of input data.

Typical cccDNA replication dynamics for each sample of parameters show a disease equilibrium state, as shown in Fig. 3(a). Copies of cccDNA appear to be in steady-state after about 12 days of infection for each set of parameters, though some cccDNA curves appear in steady-state afterwards. All cccDNA curves show a steady state at the end of the simulation (at *t* = 300 days) with substantial variation in magnitudes as shown by the histograms in Fig. 3(b); non-zero particle counts at the steady-state equilibrium of cccDNA indicates the chronic HBV infection with the immune-tolerant status of patients (Wu & Chang, 2015). Variations in the steady-state cccDNA copy levels illustrate the sensitivity of this particular model output to the range of inputs tested. Precisely which parameters hold the greatest influence we consider next.

### 3.2 Scatter plots: monotonic relationship between input and output variables

**Fig. 4:**
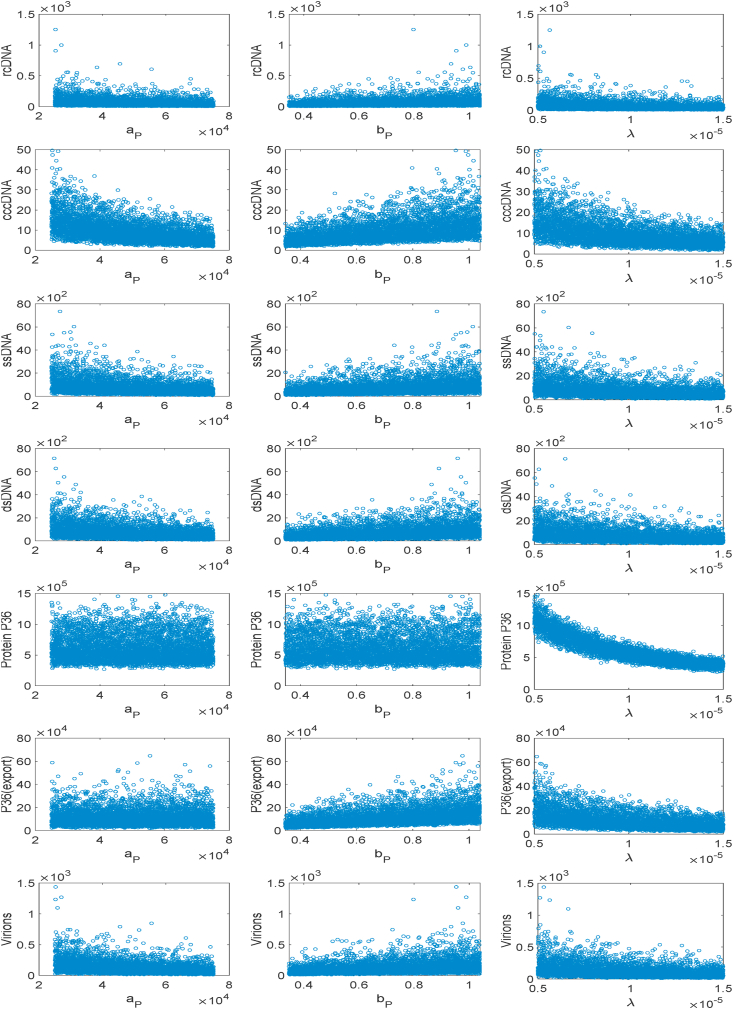
Model is simulated using Latin hypercube samples of size *N* = 5200 and the scatter plots are depicted for each population with respect to different interesting parameters to show the monotonicity of the model outputs over the parameter regime.

Simulation results of the model (Murray & Goyal, 2015) based on LHS samples of size 5200 are visualized by scatter plots (shown in Fig. 4) and they demonstrate a monotonic relationship between all output variables and input variables. These apparent monotonic relationships suggest the PRCC analysis is thus suitable for application to the HBV model utilized here. We see a wide range of sensitivities varying from strong sensitivity to negligible. In fact, all outputs except *p36* increase monotonically with the increase of *b*_*P*_ parameter values, all outputs except *p36* released from the cell decrease monotonically with the increase of *a*_*P*_ values, and the increase of *λ* contributes to a monotonic decrease of all outputs. The values of parameters *k*, *a*, *b*_*S*_ and *C*_*PE*_ contribute to the nominal change of respective outputs (results not shown). Thus, the parameters *k*, *a*, *b*_*S*_ and *C*_*PE*_ are nominally sensitive or insensitive (results not shown), and *b*_*P*_, *a*_*P*_ and *λ* are strongly sensitive to the outputs, but the sensitivity of outputs to other parameters are not observed (results not shown).

cccDNA dictates infection intensity and duration of HBV, so it is instrumental to know which parameter contributes most to the cccDNA replication. The scatter plots as shown in Fig. 4 indicate that three parameters - protein synthesis rate (*a*_*P*_), protein export rate (*b*_*P*_) and rcDNA transportation rate (*λ*) to nucleus are highly correlated with cccDNA replication within the cell. We observe here a positive correlation to protein export rate (*b*_*P*_) but a negative correlation to protein synthesis rate (*a*_*P*_) and rcDNA transportation rate (*λ*). The positive correlation of cccDNA replication to *b*_*P*_ ensures that if the rate increases, i.e. if protein p36 exports enough to maintain the protein level in the cell, then cccDNA levels double (Murray & Goyal, 2015). On the other hand, a negative correlation of cccDNA to the parameter *a*_*P*_ shows that if the values of *a*_*P*_ increase, the protein level in the cell is balanced at a constant level by the loss of it at a rate *b*_*P*_ leading to reduced transport of rcDNA to the nucleus; thus the values of cccDNA fall. The parameter *λ* exhibits similar behaviour to *a*_*P*_. We see similar results with the PRCC and Sobol index analysis discussed in the following section. Overall, we see the level of protein *p36* produced by the infected cell has an immense effect on the replication of cccDNA – and this is reflected by the model structure (Murray & Goyal, 2015). *p36* concentrations are modulated by both feedback regulation or simple export to the extracellular space; moreover, cccDNA replication is inversely related to *p36* levels and hence key to the HBV infection dynamic. For other parameters, such as *a*, *b*_*R*_, *b*_*S*_, *b*_*V*_, *μ*_*R*_, *μ*, *c*_*PE*_, and *c*_*V*_, the synthesis of cccDNA within the infected cell has a nominal (or no) effect (results not shown).

### 3.3 Time-varying sensitivity analysis

Parameter influence may vary over the temporal evolution of the model, and sensitivity analysis allows us to assess how their significance varies over the time interval (Marino et al., 2008). For both acute and chronic HBV infections, parameter influence on the model output may vary over time. Consider our results in Fig. 3 for case (a), where the curves of cccDNA copies initially increase exponentially and after a course of time they reach a steady-state for all parameter samples. After cccDNA replication starts, HBV infection turns into an acute phase during early time points, when the autoimmune system remains activated. This subsequently turns into the persistent mode of varying degrees over time for all parameter samples. cccDNA persistence after six months in the cell is considered a chronic infection: the immune system failed to clear the virus (Ganem & Prince, 2004; Smolders et al., 2020). Thus the time points at early days of infection (roughly in the acute phase) and the time points at which cccDNA levels equilibrate at the chronic phase are crucial for pathological, physiological and pharmaceutical aspects. The fortunate case of acute infection and clearance we set aside, and instead focus on parameter values leading to such chronic infection outcomes. In this regard, where cccDNA copies increase, propagate, and persist at steady state for further replication is of interest. When specific time points are not provided, temporal sensitivity analysis may identify a significant time-dependent relationship between inputs and outputs for the whole course of simulation time.

**Fig. 5:**
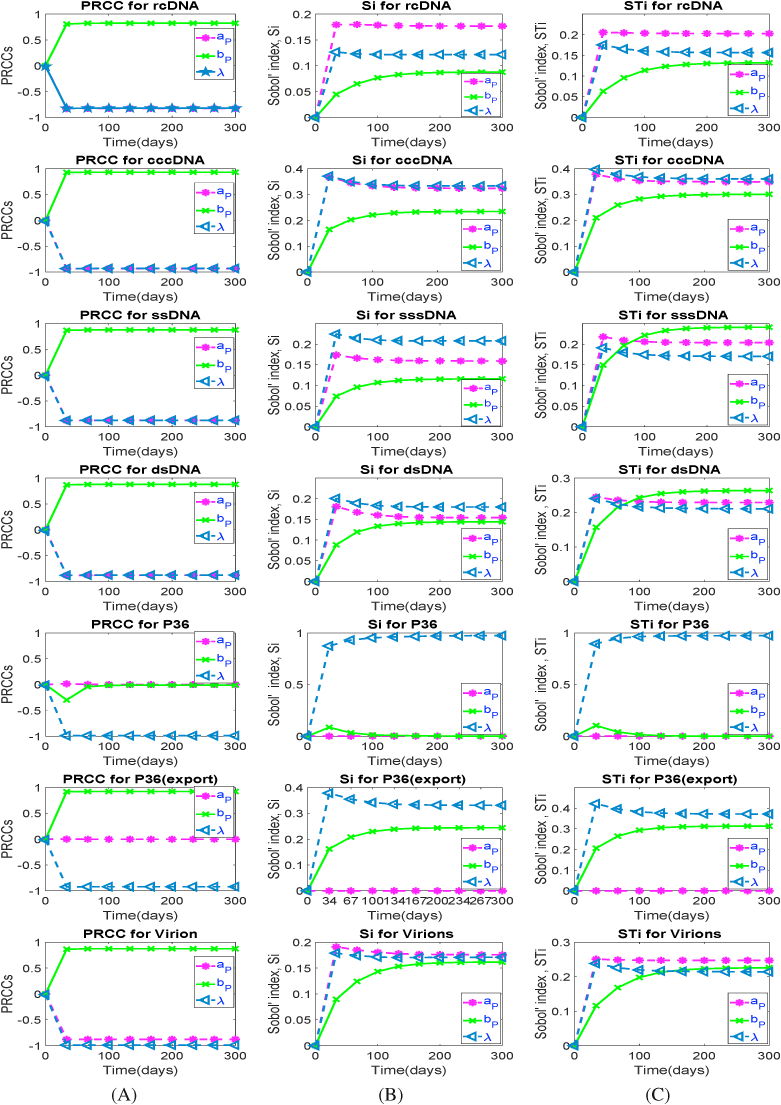
Model is simulated using samples of size *N* = 5200 and the time-varying PRCC and Sobol indices (both 1st order, Si and total effect, STi) are calculated for each population (mean value) at different time points and plotted for key parameters in the model identified to show the monotonicity of sensitivity indices over time. Recall, the PRCCs in the interval [ − 0.5 0.5] are not significant, otherwise significant. For Sobol method, parameters are significant when indices are in the interval [0.05 1.0].

In order to better capture the natural variability of HBV infection processes over time, for all parameters, we have calculated both PRCC and Sobol indices considering model outcomes at some time points chosen over the simulation time. We present results here for the parameters *a*_*P*_, *b*_*P*_ and *λ* in Fig. 5. A general trend of piece-wise linear and exponential progressions occur for the PRCC (measuring monotonic sensitivity) and Sobol (measuring contribution to variability) indices, respectively. Both indices exhibit strong sensitivity to the parameters *b*_*P*_ – the protein export rate, *a*_*P*_ – the protein (p36) synthesis rate, and *λ* – the rate of influence of protein on rcDNA to direct it to nucleus. A notable exception is that p36 is not sensitive to the parameters *a*_*P*_ and *b*_*P*_, and export of p36 is not sensitive to the parameter *a*_*P*_.

Because of strong positive correlation of *b*_*P*_ to cccDNA, as seen in Fig. 4, changes in parameter *b*_*p*_ induce a linear incremental effect on cccDNA synthesis, which means that with a linear increase of the rate of removal of protein molecules p36 created in the nucleus by cccDNA, the replication of cccDNA occurs with an overall linear increase. On the other hand, the parameters *a*_*p*_ and *λ* are strongly and negatively correlated (see Fig. 4) to cccDNA replication while the infection progresses monotonically to its steady-state (see Fig. 3).

The correlation between parameters and outputs is further annotated in the PRCC and Sobol indices analysis. The time-varying PRCCs, as seen in Panel (A) in Fig. 5, show a strong positive sensitivity of ccDNA to the parameter *b*_*p*_, after a short time of infection (early time points) and overall, progress constantly over time. Considering the impact of *b*_*p*_ – the rate at which *p36* is released into the extracellular space – we see that the positive sign of PRCC for *b*_*p*_ indicates that if parameter *b*_*p*_ is increased, cccDNA increases (and vice versa) over time (Murray & Goyal, 2015). On the other hand, the negative sign of PRCC for *a*_*p*_ and *λ* (see in Panel (A) in Fig. 5) suggests that if parameter *a*_*p*_ and *λ* decrease, cccDNA synthesis decreases (and vice versa), i.e., the level of infection caused by HBV decreases over time. Sobol indices are positive and they progress exponentially over time, indicating that the parameters - *a*_*p*_, *b*_*p*_, and *λ* are sensitive to the respective outputs with some exemptions over time.

Other parameters, such as *k*, *b*_*R*_, *a*, *b*_*S*_, *b*_*R*_, *μ*_*R*_, *μ*, *c*_*PE*_, and *c*_*V*_ remain insignificant over time with regards to the equilibrium state of cccDNA according to the PRCC method (Appendix H). Consistent with this PRCC result, these parameters according to the Sobol indices remain insignificant as cccDNA replication progresses to the equilibrium state (Appendix H).

## 4 Discussion and conclusion

Uncertainty and sensitivity analyses are capable of evaluating a model's effectiveness, and determining what factors affect model outputs. We have here investigated the sensitivity and interaction of parameters of a single-cell HBV model, illuminating model behaviour and HBV infection phenomena in a single cell. Two specific types of sensitivity analysis methods considered reliable and efficient, namely, a method based on LHS sampling (Partial rank correlation coefficient-PRCC) and a method based on variance (Sobol method) are compared. We apply them to the HBV model chosen, identify the critical parameters characterizing their influence on the model outputs, and compare the sensitivity indices for both methods. The relative merits of both approaches are further considered: each identifies similar parameter influences, yet complement with insights into their respective importance.

Both methods, for our model, are very reliable and accurate in determining the most important parameter that has the greatest effect on a specific output. Based on the PRCC indices and the Sobol first-order and total sensitivity indices, Si and STi, of the twelve parameters of interest, three parameters-*a*_*P*_, *λ* and *b*_*P*_ are found to be the leading contributors to the variance in population size of cccDNA, and nine are found to be poorly significant or even insignificant and have very low effects on the HBV model's outcomes as well as on the intensity of HBV infection in the liver. The three significant parameters are characterised by both methods as the most sensitive (treated as crucial parameters) to the outcomes of the HBV model.

Besides these three parameters-*a*_*P*_, *λ* and *b*_*P*_, PRCC shows that some other parameters are significant with a moderate effect on outputs, whereas the Sobol method is very strict to predict that only the parameters *a*_*P*_, *λ* and *b*_*P*_ are the most sensitive to the model outcomes. Therefore, the Sobol method is more robust in determining the most critical parameters compared to the PRCC method. Moreover, the Sobol indices show overall exponential progression over time, which may provide more insight on the time dependency of outputs on the parameters than PRCCs as they progress constantly over time. In the case of screening parameters during model building and simulation for a complex system, thus sensitivity analysis via the Sobol method would be precise, contributory and reliable. However, our SA results overall indicate that the PRCC and Sobol methods agree with each other.

PRCC is able to find both positive and negative correlations on model outputs. In our case, PRCC analysis reveals that the parameters, *a*_*P*_ and *λ* have a strong negative correlation to cccDNA replication, but *b*_*P*_ has a strong positive correlation with cccDNA outcome; both pieces of information are very important to take control measures against HBV viral infection and propagation in the liver. On the other hand, the Sobol indices measure just the significance of parameters with- and - without taking the effect of parameter interactions, thus it may provide reliable information for a complex system.

PRCC is efficient and brings useful insights on global sensitivity. However, PRCC requires monotonic relationships between parameters and model outputs. The Sobol method can deal with non-linear models efficiently and rigorously even though the model outputs are not monotonically related to the model parameters. In our case, scatter plots ensure that outputs are monotonic with input variables; so the PRCC method functions similarly in determining the crucial model parameters, as the Sobol method does. So, for the sensitivity analysis of this particular model, the Sobol method does not play a superior role to PRCC.

The sensitivity analysis of the single-cell HBV model using both SA methods suggests that the protein level in the infected hepatocyte (which can be controlled by adjusting the production of p36 molecules and exporting them outside the cell) is crucial for cccDNA replication. With this, three parameters *a*_*p*_ –protein synthesis rate, *λ* –P36 influence and *b*_*p*_ – protein exporting rate are termed as crucial to cccDNA replication and establishment in the nucleus, leading to chronic infection. To control chronic HBV infection, a pharmaceutical protocol for the development of HBV drug and selection of an appropriate dose of any anti-viral medicines may be built up through the adjustment of the crucial parameters.

The temporal variability of state variable response to parameters is rarely addressed for research into infectious disease modeling (Wu et al., 2013). Modelers should investigate this temporal effect as their assumptions about the value of specific parameters may be wrong, or they may misinterpret the robustness of their results if sensitivity is only tested at a single point in time. For example, if sensitivity is measured at a point after the epidemic peak, it may seem a meaningless consideration for parameters that are instrumental to the increasing epidemic curve over time. For our model, if we calculate sensitivity indices at the time point where acute HBV infection occurs, we might miss measuring the sensitivity of parameters to the chronic HBV infection (i.e. the persistence of HBV for a long time in the nuclei). The temporal sensitivity analysis of parameters to the model outputs resolves this efficiently.

HBV infection and propagation may depend on spatial aspects of liver geometry. We, therefore, want to build a spatial model of HBV (Cangelosi et al., 2017; Wang & Wang, 2007) incorporating spatial factors such as cell-cell infection and sinusoidal structures. We also suggest that the LHS-PRCC and Sobol methods are valuable for investigating parameter sensitivity in a spatial model depending on its linearity/non-linearity.

## Acknowledgements

We acknowledge the financial support from 10.13039/501100000038NSERC, Canada and Catalyst Seed grant (17-UOA-04-CSG) of the 10.13039/501100001509Royal Society of New Zealand.

## Declaration of competing interest

The authors declare that they have no conflict of interest.

## Figures and Tables

**Fig. 6:**
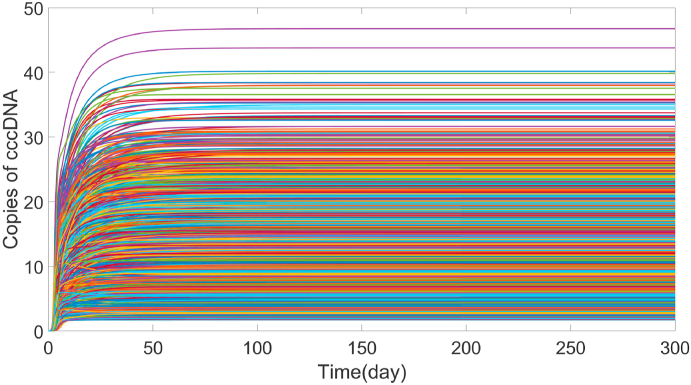
For parameter samples of size *N* = 5200 simulation is carried and corresponding cccDNA copies are plotted to show their evolution over time.

**Fig. 7:**
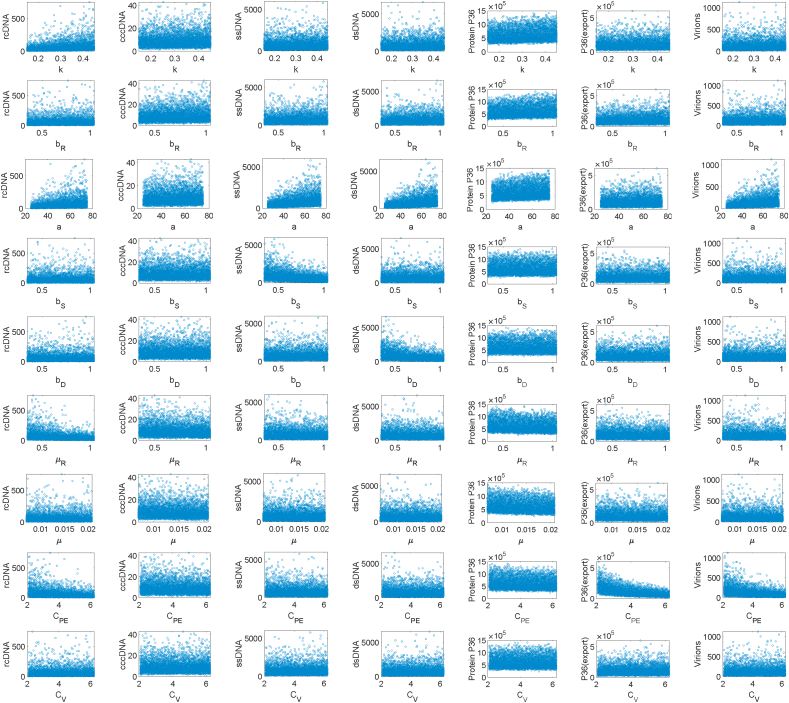
Model is simulated using Latin hypercube samples of size *N* = 5200 and the scatter plots are depicted for each population with respect to nine parameters-*k*, *b*_*R*_, *a*, *b*_*S*_, *b*_*D*_, *μ*_*R*_, *μ*, *c*_*PE*_, and *c*_*V*_ to show the monotonicity of the model outputs over the parameter regime.

**Fig. 8:**
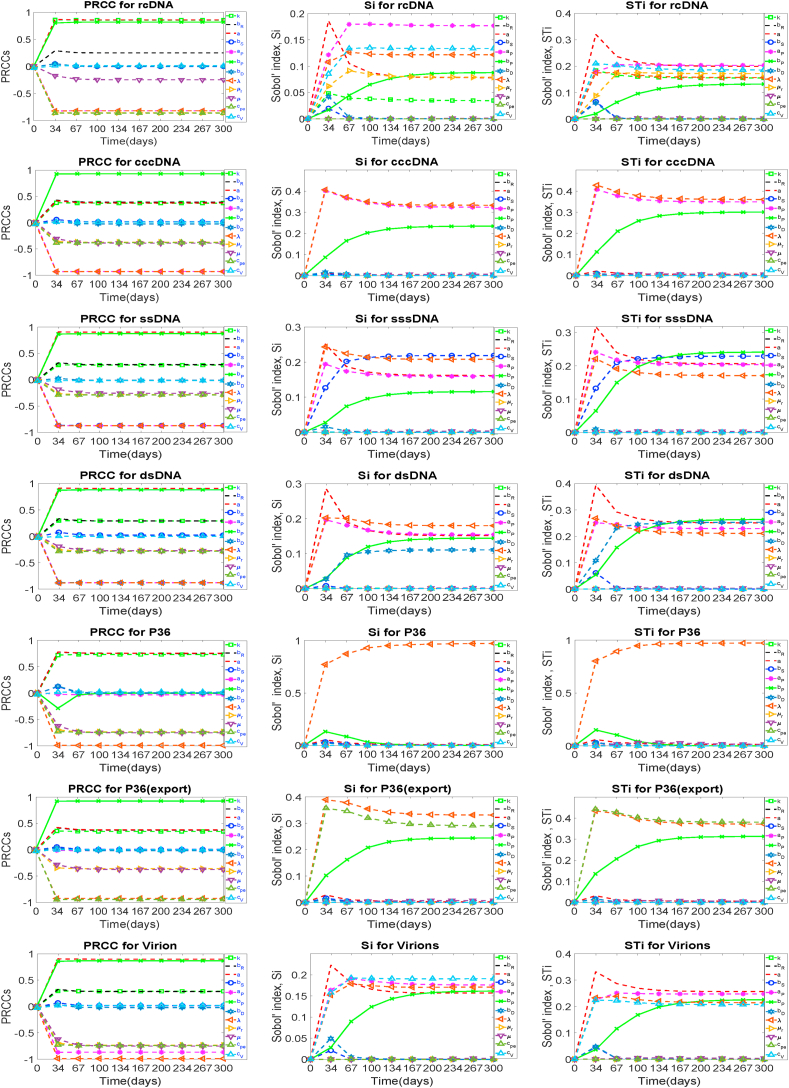
Model is simulated using samples of size *N* = 5200 and the time-varying PRCC and Sobol indices (both 1st order, Si and total effect, STi) are calculated for each population (mean value) at different time points and plotted for different parameters to show the monotonicity of sensitivity indices over time.

## Appendices

### Appendix A Latin hypercube sampling

Latin hypercube sampling is a class of stratified Monte Carlo sampling methods without replacement, and was introduced by Mckay et al. (Helton & Davis, 2003; Iman & Conover, 1982; Iman et al., 1981; McKay et al., 1979). It aims to generate samples of model inputs for investigating the sensitivity of their influence on the model outputs via PRCC analysis (see below). LHS generates a sample of size *N* for each of the *p* variables $X={\left[X_{1},X_{2},...,X_{p}\right]}^{T}$ with the probability density function *f*(*X*) in the following manner. The range of each parameter is divided uniformly into *N* disjoint intervals of equal probability 1/*N*. From each interval, one value is selected randomly according to the probability density in the interval. The *N* values of *X*_1_ are thus obtained and paired in a random manner with the *N* values of *X*_2_. These *N* pairs are then combined with the *N* values of *X*_3_ to form *N* triplets, and continued until a set of *N p*-tuples is formed. Thus, for a given sample size *N* and the number of parameters *p*, (*N*!)^*p*−1^ possible interval combinations for Latin hypercube sample are formed into a matrix sized *N* × *p*. Simulations of the mathematical model are then performed iterating over the *N* parameter suites.

### Appendix B Partial rank correlation coefficient (PRCC)

Partial rank correlation coefficient (PRCC) method is a sampling-based sensitivity analysis method that calculates the partial rank correlation coefficients for the model inputs and outputs. For two variables, *X*_*i*_ (input variable) and *Y* (output variable), a normal correlation coefficient *C* is determined as follows:

$$C\left(X_{i},Y\right)=\frac{Cov\left(X_{i},Y\right)}{\sqrt{Var\left(X_{i}\right)Var\left(Y\right)}}=\frac{\sum_{i}\left(X_{i}-\bar{X}\right)\left(Y_{i}-\bar{Y}\right)}{\sum_{i}{\left(X_{i}-\bar{X}\right)}^{2}\sum_{i}{\left(Y_{i}-\bar{Y}\right)}^{2}},$$

where *Cov*(*X*_*i*_, *Y*) is the covariance between *X*_*i*_ and *Y*, *Var*(*X*_*i*_) and *Var*(*Y*) are respectively the variance of *X*_*i*_ and the variance of *Y*; and $\bar{X}$ and $\bar{Y}$ are the sample means of *X* and *Y*, respectively. The coefficient *C* is called the Pearson correlation coefficient (*PCC*). If the data are rank-transformed, the result is called a rank correlation coefficient.

LHS-PRCC is able to draw an important conclusion regarding the importance and significance of unknown parameters of interest in contributing to the outputs of a specific model (Blower & Dowlatabadi, 1994; Zheng & Rundell, 2006). The details of the PRCC method can be found in several publications (Blower & Dowlatabadi, 1994; Marino et al., 2008; Zheng & Rundell, 2006).

### Appendix C Sobol method

The Sobol method is a variance-based GSA approach that is capable of estimating the influence of individual parameters, or a group of parameters, on the output variables of a nonlinear model (Sobol’, 1990; Sobol’, 2001). This method assembles a global index based on an ANOVA-like decomposition; the term ANOVA refers to the Analysis Of Variances. This decomposition takes place within the boundaries of the *n*-dimensional unit hypercube *I*^*n*^, where *I* is the unit interval [0, 1] and *n* is the number of input factors.

We describe the model under investigation as *y* = *f*(*x*), where *y* is the model output variable and *x* = (*x*_1_, *x*_2_, *x*_3_,…, *x*_*n*_) is a vector of input factors. We can decompose *f*(*x*) into summands of increasing dimensionality as follows:

(2) $$f\left(x\right)=f_{0}+\overset{n}{\underset{i=1}{\sum }}f_{i}\left(x_{i}\right)+\underset{1\leq i<j\leq n}{\sum }f_{ij}\left(x_{i},x_{j}\right)+\dot{...}+f_{1,2\dot{...},n}\left(x_{1},\dot{...},x_{n}\right)$$

The decomposition presented in Eq. (2) is called ANOVA-representation of *f*(*x*) if *f*_0_ is constant, and the integrals of every summand over any of its own variables must be zero, i.e.,

(3) $$\int_{0}^{1}f_{i_{1},\dot{...},i_{s}}\left(x_{i_{1}},\dot{...},x_{i_{s}}\right)dx_{k}=0,\;\;\text{for}\;\;k=i_{1},\dot{...},i_{s}$$

Equation (3) uniquely defines that the terms in Eq. (2) are orthogonal and can be expressed as integrals of *f*(*x*). In fact, the terms of the decomposition are constructed as

(4) $$f_{0}=\int f\left(x\right)dx,$$

(5) $$f_{i}\left(x_{i}\right)=\int f\left(x\right)\underset{k\neq i}{\prod }dx_{k}-f_{0},$$

(6) $$f_{ij}\left(x_{i},x_{j}\right)=\int f\left(x\right)\underset{k\neq i,j}{\prod }dx_{k}-f_{0}-f_{i}\left(x_{i}\right),$$

and so on.

We assume that *f*(*x*) is square integrable, so, $f_{i_{1}\dot{...}i_{s}}$ are also square integrable. Squaring Eq. (2) and integrating over *I*^*n*^ yields:

(7) $$\int f^{2}\left(x\right)dx-f_{0}^{2}=\overset{n}{\underset{s=1}{\sum }}\overset{n}{\underset{i_{1}<\dot{...}<i_{s}}{\sum }}\int f_{i_{1},\dot{...},i_{s}}^{2}\left(x_{i_{1}},\dot{...},x_{i_{s}}\right)dx_{i_{1}},\dot{...},dx_{i_{s}}$$

As *x* are chosen as a random point uniformly distributed within the unit hypercube *I*^*n*^, then *f*(*x*) and $f_{i_{1},\dot{...},i_{s}}\left(x_{i_{1},\dot{...},x_{i_{s}}}\right)$ are also termed as random variables and defined in the unit hypercube *I*^*n*^. From Eq. (7), we can write the total and partial variances, respectively, as follows:

(8) $$D=\int f^{2}\left(x\right)dx-f_{0}^{2}\;\;\text{and}\;\;D_{i_{1},\dot{...},i_{s}}=\int f_{i_{1},\dot{...},i_{s}}^{2}dx_{i_{1}},\dot{...},dx_{i_{s}}.$$

By squaring and integrating Eq. (2) over *I*^*n*^, the total variance is obtained as follows:

(9) $$D=\overset{n}{\underset{i=1}{\sum }}D_{i}+\underset{1\leq i<j\leq n}{\sum }D_{ij}+\dot{...}+D_{1,2\dot{...},n},\dot{...}$$

*D*_*i*_ is the fraction of variance in *f*(*x*) solely due to the variability of the *i*th parameter *x*_*i*_, and used to measure the sensitivity of *f*(*x*) to *x*_*i*_; *D*_*ij*_ is the fraction of output variance if both parameters *x*_*i*_ and *x*_*j*_ are varied, and used to measure the sensitivity of *f*(*x*) to the interaction between *x*_*i*_ and *x*_*j*_.

Now, the main effect of each factor on the model output, called the Sobol global sensitivity indices, is given as follows:

(10) $$S_{i_{1},\dot{...},i_{s}}=\frac{D_{i_{1},\dot{...},i_{s}}}{D},$$

where

(11) $$\overset{n}{\underset{s=1}{\sum }}\overset{n}{\underset{i_{1},\dot{...},i_{s}}{\sum }}S_{i_{1},\dot{...},i_{s}}=1$$

Two Sobol indices-the main effects, called the first order index - and the total effects of a parameter (Sobol’, 1990; Sobol’, 2001), are given respectively, as follows:

(12) $$S_{i}=\frac{D_{i}}{D}$$

and

(13) $$S_{T_{i}}=1-\frac{D_{∼i}}{D},$$

where *D*_∼*i*_ is the variance contributed by all parameters except *i*th parameters *x*_*i*_.

In summary, the first-order model sensitivity to each parameter *x*_*i*_ quantifies the effect if only *i*th parameter *x*_*i*_ is varied while all other parameters are fixed. The total order index is one minus the fraction of total variance attributed to *D*_∼_*i*, which represents all parameters except *i*th parameter *x*_*i*_. The total order index effectively removes parameter *x*_*i*_ from the analysis and attributes the resulting reduction in variance to that parameter. The difference between the first and total order indices is that a total-order index represents the effects of its interactions with other parameters, but a first-order index represents the effect of a single parameter variance in output. Generally, parameters with sensitivity indices greater than 0.05 are considered to be significant. The total-order sensitivity indices are greater than the first-order sensitivity indices.

### Appendix D An optimal choice for sample size

There is no *a priori* known rule determining the exact sample size for either the LHS-PRCC or Sobol method. Trial-and-error, however, proves its utility during simulations and processing of the model outputs. We do obtain a known minimum for both methods where *N* = *m* + 1 for LHS (Marino et al., 2008), and *N* = *D*(*m* + 1) and *N* = *D*(2*m* + 1) for the Sobol method. Here *m* is the number of unknown parameters of interest and *D* is the user-defined number. To obtain the correct sample size for first and second order accuracy of sensitivity indices, systematic incremental increases of sample size are made, and whether sensitivity indices will reliably capture and provide a similar set of the most significant effects is observed. If that holds for two consecutive studies of SA, there is no obvious advantage in increasing the sample size, as the conclusions will be the same. It is further noted that the sample size *N* increases if the number of model parameters increases. Usually, the higher number of samples provides better outcomes of sensitivity analysis, though the computational cost is a matter. By observation, we consider sample size *N* = 5200 for both methods in our simulation as a suitable compromise between accuracy and computational cost.

### Appendix E Computational aspects

The coupled delay different equations (DDEs) in Eq. (1) are simultaneously solved using DDE23 solver implemented in MATLAB. The solutions of the model are integrated to *t* = 300 days, where the outputs of the model remain at steady-state equilibrium. The SA technique PRCC algorithm is executed in MATLAB and an efficient SA tool SALib, a Python library, is used for calculating Sobol indices.

For SA, we sample twelve parameters- (*k*, *b*_*R*_, *a*, *b*_*S*_, *a*_*P*_, *b*_*P*_, *b*_*R*_, *λ*, *μ*_*R*_, *μ*, *c*_*PE*_, and *c*_*V*_) simultaneously, using Latin hypercube sampling and Sobol sequence techniques. The mean values of parameters, adopted from (Cangelosi et al., 2017; Murray & Goyal, 2015), provide the reasonably accurate mean values of outputs. The description of parameters and their mean values (see Value column) are given in Table 1.

The Latin hypercube sampling technique, using the uniform distribution, generates samples of parameters using parameter mean values and the desired parameter ranges, described in Table 1, as follows:

$$k=Unif\left(k_{min},k_{mean},k_{max}\right),\;\;...,\;\;c_{V}=Unif\left(c_{V_{min}},c_{V_{mean}},c_{V_{max}}\right)$$

The Sobol sequence technique also generates parameter samples using the same parameter ranges inside SALib.

### Appendix F Time-evolution of cccDNA population

We have simulated our model for samples of size *N* = 5200. For all samples, the time evolution of cccDNA copies is shown in Fig. 6. Though all cccDNA copies, initially, increases exponentially, they, eventually, show steady-state evolution as time progress.

### Appendix G Monotonic relationship between inputs and outputs

To show the monotonic relationship between input and output variables, we depict scatter plots of all outputs with respect to nine parameters – *k*, *b*_*R*_, *a*, *b*_*S*_, *a*_*P*_, *b*_*P*_, *b*_*R*_, *λ*, *μ*_*R*_, *μ*, *c*_*PE*_, and *c*_*V*_ in Fig. 7, which shows that these nine parameters do not have significant influence on cccDNA replication.

### Appendix H Time-varying PRCC and Sobol indices analysis

Time-varying PRCC and Sobol indices are plotted for all parameters, where all parameters except *a*_*P*_, *b*_*P*_ and *λ* show no influence on cccDNA replication over time, as shown in fig. 8.
